# Supplementary material for: Changes in systems thinking and health equity considerations across four communities participating in Catalyzing Communities
Source: PLoS One. 2024 Oct 23;19(10):e0309826. doi: 10.1371/journal.pone.0309826 (PMC11498710; doi:10.1371/journal.pone.0309826)
Supplement: S1 Table — Adapted from Calancie et al. (2022) [7]. *Hypothesized activities that help build participants’ systems thinking. **Evidence share varies by community (e.g., connecting early childhood education and health; promoting community health improvement through more equitable food systems). (DOCX) [file pone.0309826.s002.docx]

**S1 Table. Generalized Catalyzing Communities meeting sequence and activities across communities**

| # | Meeting Sequence | Meeting Summary | Description of Group model  builing Activity |
| --- | --- | --- | --- |
| 1 | Project Overview & Creating a Shared Vision | - Committee and research team introductions - Project overview (introduction to group model building, systems dynamics, and data collection timeline) - **Hopes and fears (group model building)** - Shared vision group discussion | **Hopes and fears:** Prompted with the question, “What are your hopes and fears for this project?” Committee members shared their personal hopes and fears for the project, and with the help of the facilitators, organized responses into themes |
| 2 | Focusing Trend (or Reference Mode), Variables Lists and How Variables Change Over Time | - Hopes and fears recap - Reference mode introduction and discussion - **Variable elicitation** - **Graphs over time (group model building)** - Group discussion | ***Reference mode:** Changemakers and the research team worked together to identify a trend that will define the scope of the system the group will explore throughout the project. Changemakers present the trend and facilitate group discussion.  ***Variable elicitation:** Stakeholders identify variables that influence the reference mode  ***Graphs over time:** Stakeholders draw how the variables they listed have changed over a specific time horizon, and project their hoped and feared trajectories into the future. We conducted variations of this exercise in each community. |
| 3 | Identifying and Connecting System Variables | - Graphs over time recap - **Connection circles (group model building)** - Group share and discussion | ***Connection circles**: Members worked in groups to draw connections between variables around a circle using arrows in order to begin seeing how variables can be connected. We did not do this activity in every community. |
| 4 | Reflecting on the Past and Sketching a Roadmap | - Reflection on prior activities (hopes and fears, graphs over time, variable elicitation, connection circles) - **Connection circles** (focused on connection between coalition’s functioning and impact) - Group share and discussion about defining success moving forward | (see GMB activity descriptions above) |
| 5 | Visualizing Systems Connections and Structures | - Introduction to causal loop diagrams - **Introduction to causal loop diagrams: Technical aspects and mechanics of drawing (group model building)** - Small group drawing of causal loop diagrams - Group share - Reaction and refinement of combined causal loop diagram as whole group | ***Causal loop diagrams**: Committee members learned how to read and create causal loop diagrams. Small groups worked together to develop causal loop diagrams to visualize connections between factors identified in previous group model building activities and identify system structures, such as feedback loops, that drive trends over time. Small groups shared their models. |
| 6 | Causal Loop Diagram Elaboration and Use for Action Planning as Systems Map | - **Research team presented integrated causal loop diagram, review of causal loop diagram, progression, summary of key feedback loops (group model building)** - Small group discussion: Is there anything missing or that should be changed? - Group share and discussion | ***Causal loop diagram elaboration:** Between sessions, the research team synthesized the models to create integrated models that they shared back with the stakeholders in subsequent meetings to get their input for model improvement.  When presented back, the facilitator explained each loop and reflected on key insights before asking the group for feedback on what is missing. Refining and elaborating the causal loop diagram(s) as a group ensures that all connections are included and that all members feel represented. In some cases, causal loop diagrams were stylized into a systems map to be primarily used as a communication tool moving forward. In other cases, causal loop diagrams were shared as is in interactive Kumu presentations or in static PDFs accompanied by narrative text. |
| 7 | Identifying Opportunities for Systems Change | - Review of refined systems map and feedback loop connections - Reflection on the importance of systems change - **Individual work to generate action ideas (group model building)** - Group share and impact-feasibility grid - Group discussion | ***Action ideas:** Action ideas that targeted specific areas of the systems map were then conceptualized using an impact-feasibility grid, a tool to guide members in formulating actionable solutions, and creating a shared understanding of potential interventions within the system |
| 8 | Prioritizing Activities for Action Ideas | - Discuss action ideas and any voting on ideas between meetings - Presentation of evidence** around top strategies, developed by research team - Group discussion of each idea and what needs to happen to move forward | N/A |
| 9 | Action Planning and Catalyzing Future Work | - Action planning continued: more structured discussion around top four action items - Introductory discussion to sustainability | N/A |
| 10 | Preparing for Sustainability of Work Going Forward | - Discussion of action strategies moving forward using the systems map - Group discussion on sustainability of work moving forward - Work groups often emerge to lead specific projects or initiatives | N/A |

*Note*. Adapted from Calancie et al. (2022) (7). *Hypothesized activities that help build participants’ systems thinking. **Evidence share varies by community (e.g., connecting early childhood education and health; promoting community health improvement through more equitable food systems).
