# Supplementary material for: Changes in systems thinking and health equity considerations across four communities participating in Catalyzing Communities
Source: PLoS One. 2024 Oct 23;19(10):e0309826. doi: 10.1371/journal.pone.0309826 (PMC11498710; doi:10.1371/journal.pone.0309826)
Supplement: S2 Table — (DOCX) [file pone.0309826.s003.docx]

**S2 Table. Categories, Codes, Definitions, and Illustrative Quotes from Participant Interviews**

| **Category** | **Code** | **Definition** | **Example Quote** |
| --- | --- | --- | --- |
| Health equity thinking | Intersectional-ity | Health equity thinking recognizes the intersecting dimensions of identity, such as race, gender, class, and sexuality, and their impact on health outcomes. It acknowledges that individuals may experience multiple forms of discrimination and disadvantage and seeks to address the unique challenges faced by marginalized and underserved populations. | “…it's understanding that who you are—like race and gender—can impact your well-being.”  “it became clear that we couldn't just focus on one factor and we have to consider how things like race and access to education all intersect and impact kids.” |
|  | Social Determinants of Health | The social determinants of health are the conditions in which people are born, grow, live, work, and age that shape their health outcomes. These factors are rooted in social, economic, and environmental conditions and play a significant role in determining an individual's overall health and well-being. This includes: SES, education, employment, neighborhood and physical environment, social support and networks, culture and access to healthcare, health behavior, etc. | “So like not just medical care; it's everything around you affecting your well-being like where you were raised and your income.”  “It’s been really helpful to thinking about upstream factors like schools, food provision, and whether parents have good jobs.” |
|  | Structural Determinants of Health | Refers to the underlying social, economic, and political structures and systems that shape health outcomes and contribute to health inequities. These structural determinants are rooted in broader societal factors and have a profound impact on individuals and communities' health and well-being. Includes economic systems, political systems, social norms and values, institutional practices, environmental factors, and historical factors. | “Children are impacted by so many institutions…structures that influence everyone’s health.”  “We’ve been talking a lot about affordable housing and how that is linked to structural racism and levels of stress for families.” |
|  | Health Disparities | Health disparities refer to differences in health outcomes or access to healthcare that exist among different populations. These disparities are based on social, economic, and demographic factors such as race, ethnicity, socioeconomic status, gender, geographic location, and other dimensions of identity. | “…they [children] have different outcomes in their health and so they often face more challenges staying healthy.”  “Kids in different neighborhoods don't get the same shot at staying healthy…” |
| Health equity action | Interventions to Reduce Deterrents to Healthy Behaviors | Health equity action involves addressing disparities in health-promoting and health-damaging exposures by minimizing the promotion of unhealthy foods or behaviors, enhancing the affordability of healthy options, and mitigating physical and social conditions that discourage healthy behaviors. | “…yeah we talked about identifying and addressing barriers to healthy behaviors. It made me reflect on how we can reduce obstacles for families, like improving access to safe places for physical activity and ensuring there are affordable options for healthier food.”  “I think my understanding has changed like to make kids healthier we have to remove barriers like making parks safer.” |
|  | Build on Community Capacity | Health equity action focuses on building on the capacity of healthcare providers, organizations, and communities to address health disparities. It may involve training healthcare professionals in cultural competency, diversity awareness, and the social determinants of health. Capacity building efforts also aim to empower communities to actively participate in decision-making processes and advocate for their health needs. | “Everyone needs the tools to address health disparities…we need additional trainings like in cultural competency…and we’ve already built so many great relationships.”  “I think our community voices were already really strong but this process helped us think more about other types of capacities we can build.” |
|  | Improve Social & Economic Resources | Health equity action emphasizes the need for targeted solutions that, while not directly health-focused, have well-documented effects on health. These solutions include efforts to alleviate poverty, enhance employment options, and improve social and housing conditions. | “We have been taking action a lot more for sustaining our efforts over a longer period of time…I think this really means addressing social and structural factors like unemployment.”  “I think we need to address the basics like job opportunities, affordable housing, and poverty. These things have a huge impact on families.” |
|  | Increase Healthy Options | Health equity action involves prioritizing interventions that align with numerous recommendations for environmental and policy changes aimed at preventing obesity in general. | “I mean you’re aware of our efforts to increase accessible and affordable healthy food options in our neighborhood.” |
| Systems thinking concepts | System structure | Childhood obesity rates are influenced by systems; Systems are composed of multiple components that are arranged in a specific way that yields a function or patterns of behavior over time | “Yeah there’s been a huge shift in our work to focus on several different parts of the child health equity system…the causal diagram shows how these structures influence child health in our community.”  “I feel like the group realized just how much child health equity is a system with parts like school policies, food access, and other things.” |
|  | Links components or pieces; recognizing interconnecti-ons | Mentions components or pieces of a system that could influence childhood obesity AND links them | “…I was saying that things like physical activity and SES are connected, and that low SES might be linked to low physical activity in some cases…and how those are part of the larger system.”  “I’m not sure but I know that the pieces we identified are connected to each other.” |
|  | Scale, multi-level | Level or scope at which observations, interactions, and phenomena occur within the system | “…or sometimes focusing too much on the individual level when we really need to be addressing the system at the community level…”  “Sometime people take too much of a top down approach…I think there’s many levels to it and we need a multi-pronged approach.” |
|  | Recognizes relationships | Connections between people/things and their committee and/or in the community | “We actually have built relationships between the committee members that still exist today and we want to make sure those carry on into the future…it’s so important for systems work.”  “But like the process has deepened relationships in the committee but also residents and other community members.” |
|  | Diffusion | Spread of ideas, influence, engagement, or other factors related to childhood obesity | “I know they have told other people about our work and what we’ve learned, and we have also disseminated information in newsletters that go out somewhat regularly.”  “We’ve noticed that some of what we’ve done together has caught on with other groups.” |
|  | Multi-sector collaboration | Working together towards a common goal related to childhood obesity, involving multiple sectors | “We actually have more sectors involved now, that I know, and it’s actually created like new projects and relationships.”  “Yeah, I think we need everyone to pitch in…different sectors contributing is important.” |
|  | Transformati-on | Opportunities to change a system by adding or removing feedback loops or by changes flows of resources, information, or material | “…it’s like fundamental change to our work and the impact we have within the community…like by addressing the connection between family resources and just like time to pay attention to your child.” |
|  | Delays | The effects of actions, interventions, initiatives, or change might not show up immediately; Implications of delays in systems | “It’s hard because we know so much of the work takes a long time to see any changes…but we hear all the time from people that we are having an impact.”  “…but so much of this work--we just don’t see improvements right away.” |
|  | Complexity | Recognition that there are many people, organizations, values, interests, etc. influencing each other and influencing childhood obesity trends broadly | “Yeah, no, it’s not simple…there are so many layers and levels.”  “It’s a mix of things like diet, exercise, stress, neighborhood safety…it’s just complex.” |
|  | Visuals | Mention using models, maps, graphs, or other visuals to better understand and communicate systems | “The CLD helped so much but it also was like, okay, we need more graphs and diagrams in our work for funding and just like for educating others.”  “Our group has used the CLD in grant writing to explain our work to funders.” |
|  | Mindsets/par-adigms | Describing the values, mindsets, goals, and motivations of the people and entities that compose and therefore influence a system | “…what do we want, what do we think we can accomplish in this work, and how do we change those things given we all want something slightly different…or view it all differently?”  “We talk about creating new programs but I think changing minds and attitudes around prevention is more important.” |
| Surface System Insights | Acknowledge-ment of System | Acknowledging that a system does exist but not explaining how its parts are interrelated | “Like child health is part of a larger system and we need to focus on that next in our work.”  “Yeah, I know that childhood obesity is like it’s own system.” |
|  | Acknowledge-ment of Components | List of components of factors of a system. Components of a system refer to the individual parts or elements that together form the overall functioning of a system. | “Yeah we identified different parts like high paying jobs, family needs, and family resources.”  “We started identifying all the parts of the system, like healthcare access, school programs, and family support, that play a role in childhood obesity” |
|  | Static Snapshot | Refers to a cognitive approach that focuses on individual events. In this mode of thinking, individuals tend to view events as discrete, independent entities with clear beginnings and endings. | “…they go to the grocery store, buy groceries, and then go home and don’t cook that much.”  “Well we’ve had community events in the past but haven’t thought about how these are connected to other aspects of health promotion.” |
|  | Linear Thinking | Perceiving events as following a linear trajectory, where one event leads directly to the next, often without considering broader context or interconnections. | “Sometimes it’s just that parents don’t have enough resources so then children don’t have access to the right food.”  “We’ve tried to improve school lunches to improve kids’ health…but we didn’t look at how to do that for specific groups of students.” |
| Mid System Insights | Components are Interrelated | Identifying this means that there is a reciprocal relationship or interaction between the components, where the output or behavior of one component influences the input or behavior of another component, and vice versa. Feedback loops create a dynamic and interconnected relationship within a system. | “Like we know that each of these pieces are connected and now we are thinking more about how they influence each other…it’s all connected.” |
|  | Where to Intervene | Mentions opportunities to make changes within a system, including which drivers are important | “…it [the CLD] helped us understand where to focus to make changes in the rest of the system…but which drivers to address is hard but now I think we have the right list.”  “Figuring out the best spots to jump in, like after-school programs or better local parks, really seemed key to thinking about which points in the system to change.” |
|  | What is Transformati-on | Opportunities to change a system by adding or removing feedback loops or by changes flows of resources, information, or material; transformation refers to a fundamental and significant change in the structure, behavior, or function of a system. It involves a shift from one state or condition to another, often with the aim of improving system performance, addressing systemic issues, or achieving desired outcomes. | “Now we consider deeper changes…it’s like turning things around, going one way of doing things to a completely different way…one goal is to just understand the system better to even know what fundamental change looks like.”  “Understanding what real change looks like, like making sure healthy habits stick around long-term, not just quick fixes, was a game-changer.” |
|  | What is Generic Structure | Recognizing systems archetypes such as success to the successful, closing a gap/goal-seeking behavior; generic structure of a system refers to the fundamental pattern or archetype that characterizes the structure and behavior of a particular type of system. It represents a common structure or set of relationships that can be found across different instances or examples of similar systems. | “…like for our community we’ve seen that there’s this pattern of racism and structural racism that is often connected to trauma and stress…and that’s not just within our CLD, we’ve seen it in other communities too.”  “Some of the evidence we reviewed I think suggested that some of the things we face in our community is actually systemic and exists in most communities and that was good to know.” |
|  | Nonlinear Relationships | Describing relationship that change over time in a non-linear way (e.g., S-shaped curve, something changes a lot then levels off, exponential growth or decay); Describe the role of accumulations or levels of materials, people, resources, or concepts (e.g. self-efficacy) in a system | “It’s like when kids engage in sports and there’s like this initial positive impact on their activity levels, and then you expect a decrease in obesity rates but it’s not like that; like increasing intensity might not lead to a decrease in obesity…impact sometimes levels out and you realize that addressing children’s health is more complex…you can’t rely on cause-and-effect.”  “Some of those graphs we did helped…like it’s slow, then fast, then levels off…I think changing things can seem like that.” |
| Deep System Insights | Connecting Systems to Emergent Behavior | Describing what systems structures influence the behavior of a specific system (e.g., identifying a reinforcing loop that has a large influence on system behavior at various points over time) | “it's getting a community garden started, and suddenly more people are involved, and it grows into this bigger thing…it's not always planned, but you can see how the little actions connect and create this positive vibe in our community from building community and educating people about local resources and building trust.”  “In our group we talk about how small changes in things like school policies can lead to access to food and then new behaviors in the system, maybe even improvements in kids’ health too.” |
|  | Explaining Leverage Points | In systems thinking, identifying and explaining leverage points within a system refers to the process of identifying strategic points or interventions within a system that can lead to significant and lasting changes in system behavior or outcomes. Leverage points are areas where a small, well-targeted intervention can create a large impact or shift in the system dynamics. | “We saw that there were two places to focus to make to most change for like a ripple effect, so we focused on building community resources as well as opportunities for physical activity.”  “Well, I do think the systems model we built helped us think about key spots to make a big difference…for us it was making sure we focus on changing policies that can help minority-owned businesses thrive.” |
|  | Identifying Boundary Conditions | Describing the effect that conditions have on dynamics within a system; Identifying when conditions have a large effect and therefore need to be accurately assessed; boundary conditions refer to the limits or constraints that define the scope or extent of a system. They delineate what is considered part of the system and what lies outside of it. Boundary conditions play a crucial role in determining the behavior and dynamics of a system. | “Like for us policy change was not out of the question but like for other communities and other systems that may be outside of their scope or their capacity to address…but for us we knew we wanted to stop at policy.”  “I think I’ve learned what our barriers are and that defining the limits of the group in changing the system helped us also think about the limits of changing the conditions of the system.” |
|  | Anticipating Systemic Implications | They can anticipate the potential consequences of actions or interventions within a system. They understand that changes in one part of the system can have ripple effects throughout the system and consider the broader systemic implications of their decisions. | “When we talked about adding parks or healthier food options, I always thought about the bigger picture. It's not just about playgrounds or lunches; it's about how these changes could ripple through our community and shape how families live and how healthy our kids grow up to be.”  “The CLD (causal loop diagram) really highlighted how if we do change this one aspect of the system we will need to think about how each of the other components will be changed.” |
