# Supplementary material for: Changes in systems thinking and health equity considerations across four communities participating in Catalyzing Communities
Source: PLoS One. 2024 Oct 23;19(10):e0309826. doi: 10.1371/journal.pone.0309826 (PMC11498710; doi:10.1371/journal.pone.0309826)
Supplement: S1 File — (DOCX) [file pone.0309826.s004.docx]

**S3 Interview protocol**

**Duration: _______**

**Introduction**

Hi, my name is [*Researcher Name*]. I would like to thank you again for agreeing to participate in this brief interview for the Catalyzing Communities to Promote Child Health Equity Study. The purpose of this interview is to hear your perspectives on childhood health and obesity in your community. It is important to note that the goal of this interview is not to critique, but rather to understand your thoughts and opinions on the topic. Throughout our conversation, when I say “obesity prevention” I am referring to activities related to childhood health and wellness: food and nutrition, access to healthy foods, active play and movement, sleep, screen time, and growth and physical development.

As I described over [*email/phone*], I would like to audio-record this interview to make sure I do not miss any of your feedback. Participation in this interview is completely voluntary, so please feel free to opt out of questions you may not want to answer. You also have the right to stop participating at any time. Your comments will be kept confidential, and only the research staff will have access to the recordings. Your name will not be included in any reports that we generate from this interview. Do you agree to be recorded? If not, I will take notes of our conversation instead, to ensure that I don’t miss any of your feedback.

*If yes:* The interview will take approximately 30 minutes. Is this ok with you?

Do you agree to be recorded?

*If no:* Ok, I will just take detailed notes of our conversation to ensure I don’t miss any of your feedback. The interview will take approximately 20-30 minutes. Is this ok with you?

Do you have any questions for me before we begin?

First, I am going to ask you about your personal beliefs related to childhood obesity prevention.

1. What are your primary concerns regarding childhood health and obesity prevention in your community?
2. In your opinion, what are two of the main causes of childhood obesity in your community?

*Probe:* Can you elaborate on why you chose those two things?

*Probe:* How do you think those things relate to childhood obesity?

1. In your own words, can you describe the relationship between obesity prevention and health equity?
2. In your opinion, what are the top two actions that should be prioritized in your community with respect to childhood obesity prevention?

*Probe:* Why did you choose to prioritize these two actions?

1. What do you view as the barriers to addressing childhood obesity in your community?

Now, I am going to ask you to think about your organization or workplace as you answer these questions on childhood obesity prevention.

1. Please rank your perception of how efforts related to childhood obesity are prioritized within your organization. You can choose from: not a priority, somewhat of a priority, or a major priority.

*Probe:* why did you rank it that way?

*Probe:* what other efforts are prioritized within your organization? Do they relate to childhood obesity?

1. Please rank your perception of how much your organization considers childhood obesity to be a problem in your community? You can choose from: not a problem, somewhat of a problem, or a major problem.

*Probe:* why did you rank it that way?

1. How has your organization influenced community awareness, policies, and/or regulations related to childhood obesity prevention?

*Probe:* What are current partnerships that your organization utilizes with respect to childhood obesity prevention?

1. Do you have any additional questions for me?

***The following questions will be asked only during the second interview.***

1. Are you exploring new roles, relationships, or actions on this topic within your work as a result of being involved with the Stakeholder Committee? Or if not, can you explain why that is?

[*if yes*] Has this exploration resulted in any course of action? If so, what? If not, why do you think that is?

1. Briefly, can you reflect on the process we worked through with the Stakeholder Committee? What did you like and not like, and what is one thing you would suggest we change if we repeat this process in the future?
2. Do you have any additional questions for me?

Thank you very much for your participation in this interview.
